# Molecular determinants of context-dependent progesterone receptor action in breast cancer

**DOI:** 10.1186/1741-7015-12-32

**Published:** 2014-02-20

**Authors:** Christy R Hagan, Carol A Lange

**Affiliations:** 1 Department of Medicine (Hematology, Oncology, and Transplantation) and the Department of Pharmacology, University of Minnesota, Masonic Cancer Center, 420 Delaware St SE, MMC 806, Minneapolis, MN 55455, USA

**Keywords:** Breast cancer, Post-translational modifications, Progesterone receptor, Signal transduction

## Abstract

The ovarian steroid hormone, progesterone, and its nuclear receptor, the progesterone receptor, are implicated in the progression of breast cancer. Clinical trial data on the effects of hormone replacement therapy underscore the importance of understanding how progestins influence breast cancer growth. The progesterone receptor regulation of distinct target genes is mediated by complex interactions between the progesterone receptor and other regulatory factors that determine the context-dependent transcriptional action of the progesterone receptor. These interactions often lead to post-translational modifications to the progesterone receptor that can dramatically alter receptor function, both in the normal mammary gland and in breast cancer. This review highlights the molecular components that regulate progesterone receptor transcriptional action and describes how a better understanding of the complex interactions between the progesterone receptor and other regulatory factors may be critical to enhancing the clinical efficacy of anti-progestins for use in the treatment of breast cancer.

## Introduction

**Figure 1:**
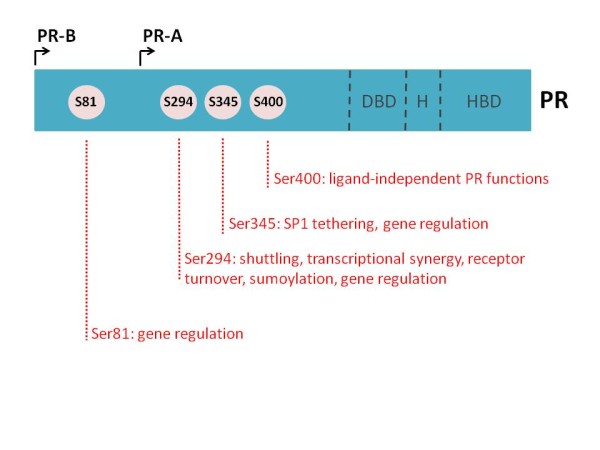
**Schematic of progesterone receptor structure and select phosphorylation sites.** Progesterone receptor (PR) isoforms A and B differ in their inclusion of an N-terminal upstream segment unique to PR-B. Both isoforms contain an identical DNA binding domain (DBD), hinge region (H) and hormone binding domain (HBD). Full-length PR-B contains 14 phosphorylation sites; serines 81, 294, 345 and 400 have known links to PR action and gene expression in breast cancer.

The mitogenic activity of estrogen is well established, but an under-studied ovarian steroid hormone, progesterone, is emerging as a primary mitogen in the breast, contributing significantly to genetic programming required for mammary stem cell self-renewal, mammary gland development, proliferation, and hyperplasia [1]. The effects of progesterone are triggered after binding of progesterone to its intracellular receptor, the progesterone receptor (PR). The PR exists in two primary isoforms, differing structurally by the inclusion of an N-terminal segment unique to the full-length isoform, PR-B [2] (Figure 1). This region, termed the B-upstream segment, is missing from the shorter isoform, PR-A [3]. The two isoforms are encoded by the same gene (regulated by distinct but tandem upstream promoters) and are most often co-expressed [4]. The PR is a member of the steroid hormone receptor subgroup of ligand-activated transcription factors within the large nuclear receptor superfamily, and is an important down-stream effector of estrogen-receptor (ER) signaling; in most circumstances, estrogen is required for robust PR expression. PR binding to DNA, either directly through progesterone response elements or indirectly through tethering interactions with other transcription factors, activates transcriptional profiles associated with mammary gland proliferation and breast cancer [5-9]. Additionally, PR binding interactions with transcriptional co-activators and repressors are critical to PR transcription factor function [10].

PRs are highly post-translationally modified, primarily through N-terminal phosphorylation (select phosphorylation sites most relevant to breast cancer biology are highlighted in Figure 1), acetylation, SUMOylation, and ubiquitination [9,11-17]. These receptor modifications dramatically alter PR function, receptor localization and turnover, and promoter selectivity. The PR can be phosphorylated basally in the absence of the hormonal ligand, but is potently modified after ligand treatment, in response to local growth factors or in a cell cycle-dependent manner [12,13,15-17] (G. Dressing and C. Lange, unpublished data). Mitogenic protein kinases - such as CDK2, CK2, and MAPK - have been shown to phosphorylate PR and subsequently modify PR action. Therefore, PR can be thought of as a ‘mitogenic sensor’ in the cell, with PR phosphorylation serving as a readout of kinase activity. Highly mitogenic environments like cancer, where kinase activities are frequently high, may be a situation where PR is persistently phosphorylated in the absence of ligand. Moreover, in this case, mitogenic signals (that is, growth factors) may diminish or replace the need for ligand, thus activating PRs inappropriately.

In addition to receiving direct inputs from protein kinases via phosphorylation, PR interacts with and activates members of cytoplasmic signaling cascades, such c-SRC [18,19]. These rapid signaling actions of PR (previously termed non-genomic actions) are independent of PR’s DNA-binding transcriptional activity [19]. However, direct PR interactions with components of kinase cascades and subsequent signaling pathway activation are highly integrated with PR genomic actions. Indeed, kinases that modify PR, as well as other growth factor-activated kinases, have been found in association with DNA-bound (that is, phosphorylated) PRs that function as part of the same transcription complexes that regulate PR-target gene promoters and enhancers [16,20,21]. Increasing knowledge about post-translational PR modifications and PR-modifying binding partners suggests that these events (such as phosphorylation, SUMOylation, and so on) are required for context-dependent activation of PR.

Understanding PR action is of great clinical significance in breast cancer, as evidenced by large-scale clinical trials conducted more than 10 years ago that demonstrated that PR actions fuel breast cancer growth. In two independent trials, women whose hormone-replacement therapy (HRT) regimens included estrogen and synthetic progesterone (that is, medroxyprogesterone acetate, norethisterone, or norgestrel/levonorgestrel) had a higher risk of developing breast cancer than women whose regimens included only estrogen and no progestins [22,23]. The results of these trials remain controversial for several reasons, including the fact that study participants were well past the onset of menopause when HRT was initiated. Additionally, although synthetic progestins clearly closely mimic progesterone *in vitro*, some synthetic progestins (medroxyprogesterone acetate) may alter androgen receptor (AR) [24] or glucocorticoid receptor (GR) [25] signaling, exhibit different half-lives, and are metabolized differently than natural progesterone, and therefore may be associated with different breast cancer risks relative to their naturally occurring counterparts [26,27]. Finally, continuous dosing of progestins as part of HRT may fail to mimic cyclical lifetime exposure to natural ligand *in vivo*. However, taken together, these landmark clinical studies implicate PR in human breast cancer development and progression, a finding that is well-supported by animal studies [28,29]. It is thus important to fully understand how activated PRs may contribute to early breast cancer progression, perhaps by driving the transition from steroid receptor (SR)-positive tumors with better clinical prognoses to more aggressive, poorer outcome SR-negative and luminal-B-type tumors.

Convincing preclinical and clinical evidence suggests that progestins increase breast cancer risk in part by driving the proliferation of early lesions [28,30-35]. Even so, at least five main sources of confusion remain regarding the role of PR actions in breast cancer (expanded on in Box 1). First, PR action is context dependent - that is, PR action differs in normal versus neoplastic tissue and according to hormone exposure (for example, in the presence versus absence of estrogen), as well as organ site (for example, proliferative in the breast versus inhibitory in the uterus). Moreover, despite convincing progestin-dependent proliferative responses in murine models [32,36,37], early reports showed that progesterone was anti-proliferative or non-proliferative in human cells [38-40]. However, recent work from the laboratory of C. Brisken [41] has shown that progesterone is proliferative in human breast tissue microstructures isolated from normal human breast specimens. Interestingly, progesterone-dependent proliferation and signaling is preserved only when the tissue architecture remains intact; human tissues (previously dissociated) grown in two- or three-dimensional cultures did not display this proliferative phenotype, suggestive of further context-dependent PR actions. Second, PR isoform-specific activities (PR-A versus PR-B) overlap but can have very disparate activities within a given target tissue and at selected gene promoters; however, despite their distinct activities, the two PR-isoforms are not distinguished clinically. Third, ligand-independent (that is, growth factor- or kinase-dependent) activities of PR are poorly understood. Fourth, the dosing (cyclical versus continuous) and source (natural versus synthetic) of ligand are likely to be key determinants of the kinetics of PR action. Fifth, although anti-progestins showed clinical promise in early clinical trials, their use was limited by liver toxicities (onapristone; [42]) largely attributable to cross-reactivity with other nuclear receptors, such as GR. This review will focus on the molecular determinants of PR’s context-dependent actions and their clinical significance. These PR actions are primarily determined by the availability of PR-binding partners and direct modifications to PR that dictate promoter selection.

### Post-translational modifications and molecular interactions alter promoter selectivity

Mounting evidence suggests that post-translational modifications of PR are key determinants of promoter selectivity and, in turn, the spectrum of target genes activated in response to ligand binding (reviewed in [43,44]). PR promoter preference is partially dictated by differences in the recruitment of PR and/or its co-activators or co-repressors to specific DNA sequences. In microarray analyses, cells expressing wild-type PR or PRs containing single point-mutations at specific phosphorylation or SUMOylation sites exhibit dramatic changes in PR-dependent gene expression, specific to precise post-translational modifications. For example, recent analyses from the Lange laboratory revealed that PR phosphorylation on serine 294 favors the subsequent deSUMOylation on PR lysine 388 [45], thereby yielding a hyperactive receptor that regulates a unique gene expression signature found in high ERBB2-expressiong tumors; this unique phospho-PR gene expression signature predicted decreased survival in patients treated with tamoxifen [9]. By contrast, a separate gene expression pattern is observed when PR is phosphorylated on Ser81 by CK2, a kinase commonly overexpressed in breast cancers; this modification is associated with the expression of gene sets involved in interferon and STAT5 signaling (discussed in more detail below) [8]. Therefore, in response to ligand, growth factor-mediated PR phosphorylation (or phosphorylation-dependent alterations of other post-translational modifications such as SUMOylation) dictates the selective expression of specific subsets of target genes and subsequently their transcriptional programs.

Target gene selectivity is achieved not only through differential recruitment of PR [8,16], but also through associated transcriptional co-activators and repressors that are critical to PR function [9,10,46]. For example, pioneer factors are specialized subsets of transcription factors that open defined regions of chromatin, making it accessible for other transcription factors, like SRs (reviewed in [47,48]). These types of factors have been identified for other nuclear receptors, such as ER and AR; however, they have yet to be identified for PR. Preliminary data suggest that FOXA1 and STAT5 may be putative pioneer factors for PR [8,49,50]; differential binding interactions between PR and these factors provide a mechanism for promoter selectivity, perhaps based on PR post-translational modifications (that is, via phosphorylation-specific interactions with pioneer factors).

Emerging evidence suggests that interactions between members of the SR superfamily is an additional regulatory step in determining target-gene specificity. Interactions between ER and AR have been the focus of recent investigations [51,52]. Recent data from the Lanari group demonstrate the existence of functional cross-talk between ER and PR; both receptors are localized together on regulatory regions of PR-target genes, such as *CCND1* and *MYC*, primarily in response to treatment with progestins [53]. Moreover, work recently published from our group suggests a complimentary story whereby ER and PR cooperate to regulate a subset of ER-target genes in response to estrogen, but fully independent of exogenously added progestin. In this case, PR-B appears to act as a scaffolding molecule for increased recruitment of signaling adaptors and protein kinases that phosphorylate ER within ER/PR-containing transcription complexes [54]. Taken together, these studies suggest that context-dependent progesterone/PR action may in part depend on the presence of other steroid hormones and their receptors. Detailed biochemical studies of steroid hormone receptor cross-talk are needed to provide a framework for a better understanding of differential hormone actions in pre- and post-menopausal conditions where endogenous hormone levels dramatically differ, as well as during breast or prostate cancer treatment with hormone-ablation therapies where closely related steroid hormone receptors (PR, GR, AR, ER) may substitute for the blocked activity of another (ER or AR).

### Progesterone receptor phosphorylation by CK2 as a paradigm for receptor modification and regulation

**Figure 2:**
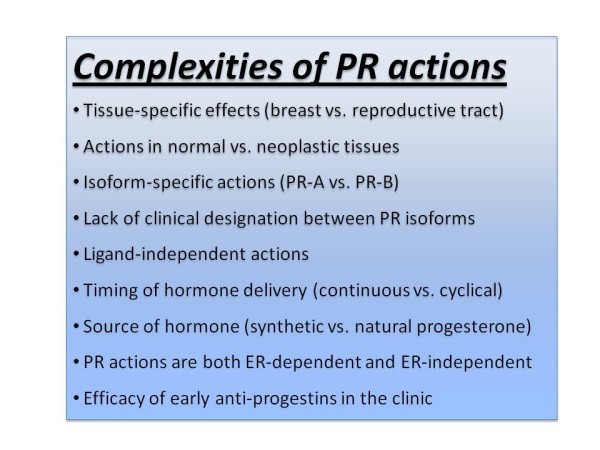
**Molecular determinants of progesterone receptor action.** Co-activators/repressors: interactions between PR and known transcriptional co-activators (for example, SRC1) and co-repressors (for example, NCOR/SMRT) are a key determinant of promoter specificity. Pioneer factors: interactions with predicted PR pioneer factors (for example, STAT5, putatively) lead to chromatin remodeling, allowing for efficient PR recruitment and subsequent target-gene transcription. Different pioneer factors would be predicted to determine differential PR recruitment. Post-translational modifications: phosphorylation (P), acetylation (Ac), ubiquitination (Ub), and SUMOylation (Sumo) primarily on N-terminal serine and lysine residues dictate receptor localization, turnover, subcellular localization, and promoter selectivity. Steroid receptor (SR) interactions: emerging evidence suggests that interactions between members of the steroid receptor superfamily (such as ER and PR) determine PR target-gene specificity. Scaffolding interactions: PR interaction with proteins acting as scaffolds (such as DUSP6) determine receptor post-translational modifications, thereby contributing to promoter selection. Cell cycle: phosphorylation on select PR serine residues and cell cycle-dependent protein complex formation determine receptor function and recruitment of PR to specific target genes.

Recent data from our laboratory characterizing PR phosphorylation on Ser81 by CK2 exemplifies how the aforementioned modifications and signaling inputs can alter PR function. CK2 is a ubiquitously expressed kinase often up-regulated in many different types of cancer, including breast [55-57]. We and others have shown that CK2 phosphorylates PR on Ser81, a site that is basally phosphorylated; however, Ser81 phosphorylation levels increase markedly in response to ligand (or when cells enter S phase in the absence of ligand) [16,58]. PR phosphorylation at Ser81 is associated with a specific gene expression profile, which is correlated with pathways altered in breast cancer, including genes implicated in mammary stem cell maintenance and renewal [8,16]. Additionally, the PR target genes whose expression require phosphorylation at Ser81 are significantly associated with interferon/inflammation and STAT-signaling datasets, a unique observation for SRs that represents a novel link between steroid hormone action, inflammation, and cancer [8]. A key target gene regulated by Ser81 phosphorylation is *STAT5* itself, and notably, JAK/STAT signaling is required for potent activation of PR Ser81-regulated genes, indicating a feed-forward mechanism for gene program activation (Figure 2). STAT5 is present, along with phosphorylated PR, on the regulatory region of *WNT1*, a key Ser81 target gene known to be involved in cancer and stem cell biology. Moreover, an in silico analysis of a publically available PR whole genome chromatin immunoprecipitation dataset reveals that there is significant enrichment of STAT5 consensus sites within PR-bound chromatin regions, indicating that STAT5 may function as a pioneer factor for phosphorylated PR (perhaps specifically when PR Ser81 is phosphorylated). These data suggest that CK2-mediated Ser81 phosphorylation of PR may activate gene expression programs involved in modulating inflammation related to breast cancer development and progression, including mammary stem cell maintenance and self-renewal.

Recent studies have defined a new mechanism by which CK2 and PR interact. Direct interaction between PR and DUSP6, a negative regulator of the MAPK pathway, is required to achieve phosphorylation on PR Ser81 [8]. This regulation occurs independently of DUSP6 phosphatase activity, suggesting that DUSP6 is acting as a scaffold for the interaction between PR and the kinase that phosphorylates Ser81, CK2. Related to this finding, an interaction between DUSP6 and CK2 has previously been identified [59]. Together, this suggests a model whereby DUSP6 binding to CK2 brings the kinase (CK2) in close proximity to its substrate (PR Ser81), allowing for efficient phosphorylation and subsequent selection of target genes within a given (that is, inflammatory, pro-growth, survival) genetic program.

Cumulatively, in this vignette describing one context-dependent scenario of PR action, there exists cross-talk between mitogenic kinases (that is, CK2 phosphorylation of PR Ser81), MAPK pathway components (that is, DUSP6 interaction with PR is required for Ser81 phosphorylation), phosphorylation-dependent gene regulation (that is, Ser81 phosphorylation is required for PR recruitment to specific subsets of PR target genes), and putative phosphorylation-specific interactions with a pioneer factor/co-factor (that is, JAK/STAT-dependence of PR Ser81-regulated gene expression). PR phosphorylation by CK2 on Ser81 is an exemplary case study of how the molecular determinants of PR action differentially determine receptor function in breast cancer models (Figure 2).

### Progesterone receptor clinical significance in breast cancer

Luminal breast tumors are characterized by their expression of ER and PR, both of which are good prognostic markers for predicted response to endocrine therapies. Interestingly, analysis of The Cancer Genome Atlas data for the luminal A/B subtype of breast tumors reveals that heterozygous loss of the PR locus occurs in 40% of luminal tumors, while 25% of luminal tumors are also heterozygous for the ER locus. However, these tumors are overwhelmingly ER-positive and largely respond well to ER-targeted therapies [60]. Interestingly, PR and ER copy number is often correlated in individual tumors; tumors with altered copy numbers for ER are likely to have changes in PR copy number. Despite these genomic alterations, both PR and ER mRNA levels are similar in luminal tumors that are diploid versus those that have lost an allele at these loci. Thus, gene copy number may not be a robust measure of the functional (that is, protein) readout for these steroid hormone receptors and should be interpreted with caution. Moreover, complex intra- and inter-tumoral heterogeneity may be reflected in analyses of genomic copy number. Because PR-positive cells release pro-proliferative factors (that is, PR target-gene products) that induce paracrine signaling, a small percentage of PR-positive cells within an individual tumor could have significant effects on tumor stem cell maintenance and/or tumor growth and progression. This is a complex situation that makes PR loci genomic heterozygosity difficult to interpret. Cumulatively, these data underscore the need to gain a much better understanding of PR signaling within the clinical context.

HRT clinical trial data (discussed above) suggest an important role for progestins and PR as drivers (that is, tumor promoters) of breast cancer cell growth. Progesterone-dependent expression of secreted paracrine factors is required for self-renewal of (PR-null) stem cells in the normal mammary gland [32,37] (see below). PR target genes include soluble factors known to modify cancer stem cells (WNT1 and RANKL). However, the role of PR target genes in the maintenance or expansion of cancer progenitor or stem cells is currently unknown. While a minority of normal (non-pregnant) breast epithelial cells contain steroid hormone receptors, the majority of luminal breast cancers express ER and PR (discussed above); heterogeneous cells within the breast may contain both ER and PR, only ER, or only PR [61]. Interestingly, very few somatic mutations have been identified in ER [62] or PR. With regard to PR, isolated genetic polymorphisms linked to breast and reproductive cancers appear to increase levels of PR-B isoform expression, rather than affect PR transcriptional activity [63-65]. Additionally, the PR-A promoter is more frequently methylated (that is, silenced) relative to the PR-B promoter in advanced endocrine-resistant breast cancers [66]. These data imply that genetic alteration of PR itself is usually not sufficient to promote tumorigenesis. Alternatively, we propose that oncogenic mutations that drive signaling pathways provide the context for heightened ER and PR transcriptional activity. For example, high levels of kinases, such as CK2, CDKs or MAPKs, may induce persistent progesterone-independent phosphorylation of PR-B on serines 81 or 294, respectively, thereby leading to activation of phospho-isoform-specific transcriptional programs shown to be significantly altered in luminal breast cancer [8,9]. Therapeutic strategies that target receptor-modifying protein kinases (that is, anti-CK2, CDK2 or MAPK) and/or their transcriptional co-factors (that is, STATs, AP1, SP1, FOXO1, FOXA1) are likely to be very successful at treating breast cancer and must remain a direction of robust exploration within the SR field.

Historically, clinical testing of anti-progestins has been limited [42,67-70]. The results of a clinical trial released in 1999 showed promise for anti-progestins as front-line breast cancer endocrine therapy [42]. Although patient accrual in this study was small (19 patients), 67% of patients achieved tumor remission when treated with onapristone, a PR type I antagonist that blocks PR binding to DNA, as front-line endocrine therapy for locally advanced or primary breast cancer [42]. Liver function test abnormalities were seen early in this trial, and for that reason new patient accrual was stopped. These liver-associated effects were likely due to inhibition of GR, a closely related SR. The clinical efficacy of lonaprisan, a type III PR antagonist that promotes PR repression through the recruitment of transcriptional co-repressors (while maintaining DNA binding), was measured in a phase II study as second-line therapy for PR-positive breast cancer [70]. The results from this trial were disappointing, and the trial was terminated before full patient accrual. Although a small percentage (14%) of patients achieved stable disease, no patients achieved complete or partial responses. This trial likely failed for a number of reasons, including lack of patient classification, patients having previous exposure to endocrine therapies, and a lack of mechanistic understanding of PR inhibitor action and isoform specificity. Notably, clinically used anti-progestins that target the ligand-binding domain of PR may fail to block ligand-independent actions of PR (discussed above).

Renewed optimism for the use of anti-progestins to prevent or inhibit breast cancer growth is provided by more recent preclinical studies of anti-progestins in murine mammary tumor models. In a dramatic example, treatment of nulliparous *Brca1/Trp53*-deficient mice with mifepristone, a PR antagonist, completely inhibited the formation of mammary gland tumors normally observed in virgin mice [71], perhaps via modulation of the stem cell compartment [30,32]. Newer, highly selective anti-progestins, which are currently in development by several pharmaceutical companies, may increase the clinical utility of anti-progestins in breast cancer prevention and treatment and is an area of renewed research interest. Notably, many patients that relapse while on tamoxifen therapy retain expression of PR, underscoring the clinical significance of considering PRs as potentially acting independently of ER in the context of breast cancer progression during estrogen ablation (that is, PR expression is most often used clinically as a measure of ER function) [72,73]. Based on our current understanding of ligand-dependent and ligand-independent (kinase-induced) PR actions, classification of patients based on gene-expression profiling could better identify the subpopulation of patients that would respond well to selective anti-progestins. In addition, cross-talk between ER and PR (or AR), and growth-factor signaling pathways (discussed above) is a likely confounding component of development to endocrine-resistant disease, and should therefore be considered (for example, via the use of pathway-specific gene biomarkers) when selecting anti-progestins as potentially beneficial front-line or second-line therapy [74-76].

As mentioned above (and in Box 1), the clinical significance of PR isoforms is likely vastly under-appreciated. In mammary tissue, PR exists as two primary isoforms, PR-A and PR-B. Although PR-B is required for mammary gland development and PR-A for uterine development, these isoforms are most often co-expressed in the same tissues, typically at a ratio of 1:1. Single isoform expression in tissues is rare [77-79]. Interestingly, in pre-neoplastic lesions and samples from patients with breast cancer, this balanced A:B ratio is often altered, frequently due to apparent loss of PR-B [78,80]. Cumulative data from the Lange laboratory has revealed that this imbalance may be explained by phosphorylation-dependent turnover of transcriptionally active PR-B receptors relative to more stable and less active PR-A receptors. PR-B but not PR-A undergoes extensive cross-talk with mitogenic protein kinases [8,16,45,81,82]. Thus, PR-B is heavily phosphorylated in response to ligand or via the action of growth factors, and although this isoform-specific phosphorylation (on PR-B Ser294) is linked to high transcriptional activity, it is also coupled to rapid ubiquitin-dependent turnover of the receptor; regulated PR-B turnover is tightly linked to transcriptional activity (that is, stable non-degradable mutants of PR are poor transcriptional activators) [83,84]. Of note, this phosphorylation event (PR-B Ser294) has been detected in a subset of human tumors [9]. Therefore, loss of PR-B, as measured by protein levels in clinical immunohistochemistry tests or western blotting may actually reflect high PR-B transcriptional activity coupled with rapid protein turnover; peak PR target-gene expression (mRNA) is coincident with nearly undetectable PR protein in experimental models [85]. Mouse models (mammary gland) predominantly express PR-A prior to pregnancy. In humans, normal mammary gland function may rely upon balanced expression of the two PR isoforms. Unfortunately, current immunohistochemistry clinical testing for PR in breast cancer samples does not differentiate between PR-A and PR-B isoforms. Because an imbalance between the two isoforms appears to be linked to cancerous phenotypes, clinical isoform distinction may have great diagnostic potential and should be considered as part of routine luminal cancer work-up.

Emerging data linking progesterone regulation to the expansion of the mammary stem cell compartment highlight the role that PR and progesterone may play in early events in breast cancer. Recent seminal work in murine models has shown that progesterone can induce the rapid expansion of mammary stem cells, a population of SR-negative (that is, ER- and PR-negative) cells located in the basal epithelial compartment of the mammary gland [32,37]. Because these cells are PR negative, this expansion likely occurs through the production of paracrine factors secreted by neighboring or nearby PR-positive luminal epithelial cells. Progesterone-dependent expansion of the mammary stem cell population is mediated by key PR-target genes, including *RANKL* and *WNT4*[32,37]. Brisken and colleagues have shown that progesterone-dependent control of RANKL expression in human tissues is dependent on intact breast tissue microstructure, and have confirmed that RANKL is required for progesterone-induced proliferation [41]; estrogen is a permissive hormone (for PR expression) in this context. Interestingly, PR-dependent RANKL expression requires STAT5A [50]. This observation is similar to what has been published for PR regulation of WNTs [8], highlighting an emerging role for co-ordinate STAT5/PR regulation of select subsets of PR-target genes related to proliferation and stem cell self-renewal (see above). Moreover, a PR-positive subpopulation of mammary gland progenitor cells has been recently discovered [61], challenging the current dogma that mammary gland precursors are strictly SR-negative. These exciting findings suggest that this long-lived population of cells, one that is exquisitely sensitive to mutagenic events, can expand in response to progesterone in both a paracrine and autocrine fashion [36]. Notably, these PR-positive mammary stem cells are devoid of ER protein or mRNA expression, further underscoring the need for understanding PR action as independent of ER in this context.

## Conclusions

Recent clinical and preclinical studies clearly demonstrate the significance of fully understanding the determinants of context-dependent PR action. They not only challenge the current clinical diagnostic paradigm in which PR is only used as a marker of ER transcriptional activity, but also support a renewed interest in understanding PR as a driver of breast tumor progression and thus a potentially very useful target for improved breast cancer therapy [1,86]. In this review, we have highlighted the concept that gene-expression analyses linked to PR actions suggest different transcriptional programs are activated in response to specific post-translational modifications (phosphorylation events) and protein-protein interactions. Although these unique PR gene signatures highlight functional differences between modified PRs and their components, the overlap between these (predominantly proliferative) programs supports a strong role for PR in early tumor progression toward more aggressive cancer phenotypes, and in some cases, even highlights a phospho-PR gene signature associated with poor response to endocrine treatment [9]. Therefore, gene signatures that define PR action will likely provide a useful paired diagnostic for clinically applied selective anti-progestins. We conclude that PR function is highly dependent on the molecular context, which is defined by such factors as protein kinase activity (as a major input to receptor post-translational modifications), co-factor availability, and the presence of progesterone and other steroid hormone levels and receptors (Figure 2). Future therapeutic approaches should consider targeting receptor-modifying activities in place of or in conjunction with anti-hormone therapies. With progesterone emerging as the primary mitogen in the adult breast (wherein estrogen is permissive for PR expression), understanding PR function and identifying or targeting modifiers of PR action are of critical importance to advancing the treatment of breast cancer.

### Box 1: Complexities of progesterone receptor actions.

• Tissue-specific effects (breast vs. reproductive tract)

• Actions in normal vs. neoplastic tissues

• Isoform-specific actions (PR-A vs. PR-B)

• Lack of clinical designation between PR isoforms

• Ligand-independent actions

• Timing of hormone delivery (continuous vs. cyclical)

• Source of hormone (synthetic vs. natural progesterone)

• PR actions are both ER-dependent and ER-independent

• Efficacy of early anti-progestins in the clinic

## Abbreviations

AR: androgen receptor; ER: estrogen receptor; GR: glucocorticoid receptor; HRT: hormone-replacement therapy; PR: progesterone receptor; SR: steroid receptor.

## Competing interests

CAL receives consulting income from Arno Therapeutics, Inc. This interest has been reviewed and managed by the University of Minnesota in accordance with its Conflict of Interest policies. CRH declares that she has no competing interests.

## Authors’ contributions

CRH and CAL together led the initial design and conception of the manuscript. CRH led the writing of the first and all subsequent drafts of the manuscript. CAL contributed significant written and editorial inputs to the manuscript at every stage. Both authors read and approved the final manuscript.

## Authors’ information

CAL joined the University of Minnesota (Departments of Medicine and Pharmacology) faculty in 1999. Her research is focused on steroid hormone action in breast cancer progression. Her laboratory studies the role of cross-talk between growth factor-mediated signaling pathways and steroid hormone receptors, using the human progesterone receptor as a model receptor. CAL holds the Tickle Family Land Grant Endowed Chair of Breast Cancer Research at the University of Minnesota. She is the Director of The Cancer Biology Training Grant (T32) and the Cell Signaling Program Lead within the Masonic Cancer Center. CAL is Editor-in-Chief of the journal *Hormones and Cancer* (jointly held by The Endocrine Society and Springer). CRH is a senior post-doctoral fellow in the laboratory of CAL.
